# Occupancy Estimation from Blurred Video: A Multifaceted Approach with Privacy Consideration

**DOI:** 10.3390/s24123739

**Published:** 2024-06-08

**Authors:** Md Sakib Galib Sourav, Ehsan Yavari, Xiaomeng Gao, James Maskrey, Yao Zheng, Victor M. Lubecke, Olga Boric-Lubecke

**Affiliations:** Department of Electrical & Computer Engineering, University of Hawai’i at Manoa, Honolulu, HI 96822, USAyaozheng@hawaii.edu (Y.Z.); lubecke@hawaii.edu (V.M.L.)

**Keywords:** occupancy counting, deblurring, deep learning, machine learning, image processing, privacy

## Abstract

Building occupancy information is significant for a variety of reasons, from allocation of resources in smart buildings to responding during emergency situations. As most people spend more than 90% of their time indoors, a comfortable indoor environment is crucial. To ensure comfort, traditional HVAC systems condition rooms assuming maximum occupancy, accounting for more than 50% of buildings’ energy budgets in the US. Occupancy level is a key factor in ensuring energy efficiency, as occupancy-controlled HVAC systems can reduce energy waste by conditioning rooms based on actual usage. Numerous studies have focused on developing occupancy estimation models leveraging existing sensors, with camera-based methods gaining popularity due to their high precision and widespread availability. However, the main concern with using cameras for occupancy estimation is the potential violation of occupants’ privacy. Unlike previous video-/image-based occupancy estimation methods, we addressed the issue of occupants’ privacy in this work by proposing and investigating both motion-based and motion-independent occupancy counting methods on intentionally blurred video frames. Our proposed approach included the development of a motion-based technique that inherently preserves privacy, as well as motion-independent techniques such as detection-based and density-estimation-based methods. To improve the accuracy of the motion-independent approaches, we utilized deblurring methods: an iterative statistical technique and a deep-learning-based method. Furthermore, we conducted an analysis of the privacy implications of our motion-independent occupancy counting system by comparing the original, blurred, and deblurred frames using different image quality assessment metrics. This analysis provided insights into the trade-off between occupancy estimation accuracy and the preservation of occupants’ visual privacy. The combination of iterative statistical deblurring and density estimation achieved a 16.29% counting error, outperforming our other proposed approaches while preserving occupants’ visual privacy to a certain extent. Our multifaceted approach aims to contribute to the field of occupancy estimation by proposing a solution that seeks to balance the trade-off between accuracy and privacy. While further research is needed to fully address this complex issue, our work provides insights and a step towards a more privacy-aware occupancy estimation system.

## 1. Introduction

Residential and commercial buildings use large quantities of energy to maintain thermal comfort, visual comfort, and indoor air quality for their occupants. However, many heating, ventilation, and air conditioning (HVAC) systems within modern buildings run on fixed schedules and assume maximum occupancy rather than actual usage, leading to high energy costs and over-conditioned space. This has made buildings one of the fastest-growing energy consumers in recent years, responsible for more than 30% of worldwide electricity and natural gas usage [1]. To minimize energy waste, several fine-grained building energy management systems (BEMSs) have been proposed recently to minimize energy waste caused by fixed scheduling of HVAC and assumption of maximum occupancy. These systems use contextual information, such as occupancy information, in addition to traditional environmental parameters, such as temperature and humidity, for dynamic HVAC control. Using low-cost infrared and magnetic sensors, Agarwal et al. developed a BEMS system that collects fine-grained occupancy information to modify the HVAC load. Using this type of occupant information, the system was able to save 10–15% of the energy used in the pilot [2]. Using a BEMS that recognizes occupants’ long-term presence patterns, Yang and Becerik-Gerber found that the HVAC system might save up to 9% [3]. Using video data and CO_2_ sensors, Wang et al. [4] suggested a predictive algorithm for HVAC control. In buildings, they exhibited a 40% reduction in energy use without sacrificing thermal comfort or air quality. The operation time of lighting systems is also determined by the knowledge about occupancy [5,6]. Occupancy-based lighting control, as proposed by Leephakpreeda et al. [7], has the potential to reduce the energy consumption of lighting systems by up to 75%, according to their findings.

Various types of sensors have been used to accurately estimate and detect building occupancy in a variety of applications. By detecting changes in temperature patterns caused by the movement of objects, passive infrared (PIR) sensors are utilized in [8,9,10] to identify the presence of people and to estimate the number of people [11,12]. The ultrasonic sensor [13] is another motion-based occupancy detection technique that uses the Doppler effect to detect the movements of people. Using the acoustic properties of the sound produced by human activity, microphone sensors [14] can detect the presence of people. These sensors are dependent on human movement and actions and, therefore, have limitations when it comes to identifying stationary objects. Several approaches [15,16] based on environmental characteristics (sound, temperature, pressure, humidity, and CO_2_ concentration) have been presented to estimate the number of occupants within an enclosed space. To improve the estimation performance of such methods, sensors of diverse parameters must be coupled. Their real-time performance is also affected by another limitation: delayed estimation. Additionally, because of the widespread availability of cellphones, the researchers proposed the utilization of Wi-Fi [17] and Bluetooth [18] signals for occupant estimation. The most significant disadvantage of both systems is that the occupants must have their devices turned on, which is also a disadvantage of the RFID tag-based method [19]. Researchers have also designed thermal-imaging-based occupancy counting systems [20] but thermal imaging has limitations in terms of low spatial resolution and contrast compared to visible light cameras, limiting its ability to precisely identify small or distant objects [21].

Occupancy can also be detected by analyzing image/video data [22]. The excellent precision of cameras makes them popular for estimating and detecting the presence of people in buildings. Floret and colleagues developed a method to estimate the locations of all indoor individuals, which can be used to determine the number of people who are present in a building [23]. They used a multi-camera system to achieve this. Vision-based occupancy estimation and detection systems were proposed by Benezeth et al. [24]. Although the accuracy of their methods was better, the number of occupants was significantly lower. For the purpose of creating an occupancy model, Erickson et al. [25] used cameras to gather data on building occupants and implemented background subtraction to identify images containing people. They then incorporated the established occupancy model into building energy management systems in order to save energy consumption. The scope of such a method is limited, as the background subtraction method fails to detect static objects and the counting process adopted here is manual. Occupancy estimation systems based on vision cameras at the entrances and within a room have been presented by Liu and others [26]. The room’s occupancy was estimated using a two-stage static detector to detect human heads in rooms and the occupancy at the entrances was detected using a motion-based approach. They employed a dynamic Bayesian network technique to combine the findings of the room’s occupancy estimation with those of the entrances. In rooms with multiple entrances, accurately counting occupants with such a method becomes a challenging and expensive task, as it requires installing cameras at each entrance and coordinating readings from all cameras. To recognize the presence of a human head, the authors in [27] employ a cascade classifier, which consists of a pre-classifier, a primary classifier, and a clustering analyzer. An experimental study found that occupancy measurement is 95.3% accurate. The experiment was performed on a dataset of surveillance videos recorded in a typical office environment. The number of people varied from 0 to 12, which is comparatively small. With the use of unsupervised image processing techniques, Petersen et al. [28] developed an occupancy estimate system that relied on a Kinect camera installed at the room’s entrance to count the number of people that entered and left the room. In order to monitor all of the entrances to all of the rooms in a big building at once, the system requires a Kinect camera and a powerful PC for each room for each entrance, if the room has multiple entrances. However, this would need a significant expenditure for research purposes. Tomastik et al. [29] developed a non-linear stochastic state-space traffic model of occupants using the output of the video camera which is processed by some real-time object detection algorithm. In [30], they applied a deep learning method to classify the images into two classes, occupied or not occupied, but did not count the number of occupants. Generally, with cameras, an indoor occupancy counting system with high accuracy can be designed. Hence, for many other occupancy estimators, cameras were commonly used to obtain the labeled data and ground truth.

The methods of occupancy estimation using cameras often provide relatively accurate results, but those algorithms also suffer from some issues, such as occlusions due to the comparatively large number of occupants, computational complexity, and the influence of illumination conditions. Most importantly, none of the image/video processing-based occupancy counting methods has addressed privacy concerns to the best of our knowledge. It is critical for a camera-based occupancy counting method to adhere to digital privacy laws and safeguard the identities of people in indoor spaces. In this work, our primary aim was to tackle the privacy concerns associated with camera-based occupancy estimation. To address the privacy issue, we intentionally blurred the video frames by changing the focal length of the camera. Furthermore, we aimed to estimate occupancy from a diverse dataset that included various crowd types, such as small, large, moderate, dense, sparse, moving, and still. Our goal was to develop a method that could accurately estimate occupancy from these challenging blurred videos while simultaneously maintaining the visual privacy of the occupants. Our contributions can be summarized as follows:

1. Developed a motion-based technique for occupancy counting from blurred video frames that is not affected by blur and can be applied directly to the blurred frames, thus inherently preserving privacy.
2. Developed motion-independent techniques for occupancy counting, including detection-based and density-estimation-based methods.
3. Proposed two different deblurring methods to improve the accuracy of downstream detection and density estimation models in motion-independent techniques:
  (a) The first method is based on the Lucy–Richardson algorithm, but unlike the original approach, the choice of blur radius is informed by the presence of blur in the image.
  (b) The second method utilizes a U-Net architecture in an end-to-end fashion, trained on synthetically blurred crowd images.
4. Conducted an analysis of the privacy implications of the occupancy counting system by comparing the original, blurred, and deblurred frames using metrics such as blur extent, structural similarity, and perceptual difference. The results showed that the deblurred frames used in the motion-independent approaches still maintained some level of visual distortion, providing a degree of privacy protection, even though it was not the primary design goal.

A comparison of different existing approaches and our proposed occupancy estimation approach is shown in Table 1.

**Figure 1:**
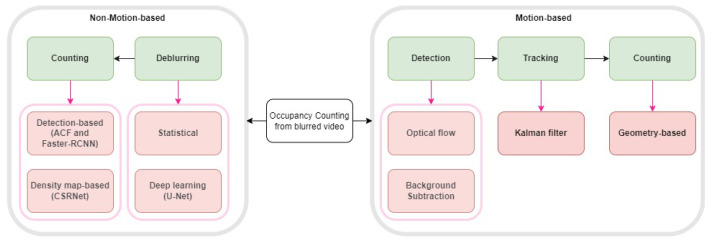
Methodology.

The paper is organized as follows: Section 1 presents the background and motivation of our work. Section 2 describes the data we worked on and the facility from which we collected the data. Section 3 briefly describes our solution approaches (Figure 1 is the graphical representation). Section 4 describes how motion information can be used to detect, track, and count the number of occupants. This section represents the usage of background subtraction and optical flow estimation to detect motion, followed by Kalman-filtering-based tracking and geometry-based counting. Section 5 presents the deblurring process, which is the first stage of our learning-based counting methods. It describes statistical and deep-learning-based deblurring techniques. This section also illustrates the second stage, which is the counting. Here, we show the application of machine and deep-learning-based detection and deep-learning-based density estimation algorithms to count the number of occupants in the deblurred frames. Section 6 compares our different proposed techniques and also discusses the effects of deblurring on occupants’ privacy. Section 7 concludes this work.

## 2. Problem Statement

### 2.1. FROG Building

**Figure 2:**
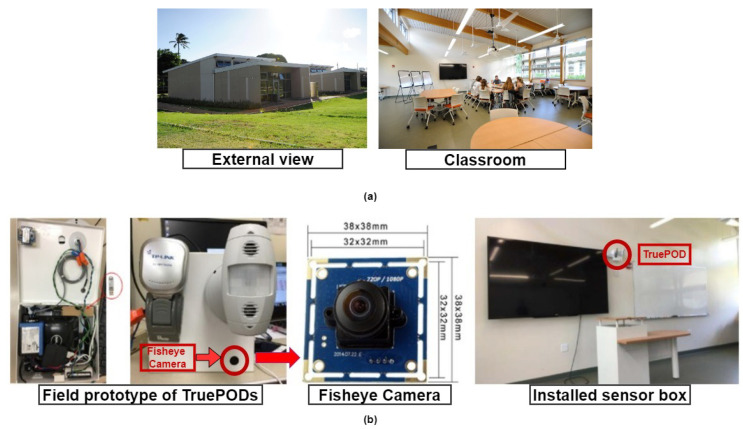
(**a**) Flexible-response-to-ongoing-growth (FROG) buildings; (**b**) data collection system in FROG building.

The dataset for this work has been collected from sustainable, energy-efficient flexible-response-to-ongoing-growth (FROG) buildings (Figure 2a) located at the University of Hawaii at Manoa (UHM) campus and managed by Hawaii National Energy Institute (HNEI). These two 1428-square-foot buildings are part of a larger research program intended to evaluate the performance and integration of a range of technologies that includes energy efficiency, storage, and renewable energy systems. The FROG buildings are utilized as classrooms for the K-12 University Lab School (ULS) in the mornings and UHM in the afternoons and evenings. Designed to be net-zero energy, both buildings are equipped with environmental and energy sensors for advanced monitoring, using a real-time dashboard that illustrates current and past operating conditions such as temperature, CO_2_ levels, illumination, humidity, and energy use by different loads like lighting, ceiling fans, air conditioning, and plug loads [31]. All of this information is being gathered with the purpose of being used to conduct research on energy-management systems. Several studies [31,32,33,34,35,36] have been and are being performed on FROG data to detect occupancy, motion, and also estimate the number of occupants and direction of arrival using Doppler radar. Our target was to provide standard reference counts to other Doppler-radar-based counting methods.

### 2.2. Data Collection and Analysis

A field prototype (Figure 2b) was developed for occupancy sensing and counting with a camera and custom-built 2.4 GHz radar [32] in conjunction with common occupancy sensors. However, for the convenience of synchronizing data from different sensors into the system, stand-alone sensors, including a Leviton occupancy sensor unit, thermometer, radar, and out-of-focus fisheye camera, are installed within our deployed sensor in the field testing sensor box, instead of using the embedded building sensors. The sensor box is shown in Figure 2a. This field prototype includes a mini PC for data recording, storage, remote monitoring, and control; a USB DAQ data acquisition device; a custom-built 2.4 GHz radar; a hybrid occupancy sensor (PIR/US) unit; a thermometer for temperature monitoring inside the sensor box; and a fisheye camera (lower right corner of the sensor box) (Figure 2b). Data were collected for occupancy count algorithm testing and optimization in an FROG building for four months. To satisfy the requirements of a wide angle of view without causing a privacy concern, a fisheye camera was adopted in the field test. The camera uses a fisheye lens that produces strong visual distortion to create a wide panoramic (180-degree field of view) or hemispherical image. The focal length of the camera is changed to make the image out of focus and blurred, and thus, it is difficult to recognize individuals. The prototype is mounted in the middle of the front wall of the 1428-square-foot classroom at a height of 2.2 m above the floor to achieve coverage of the whole classroom building.

**Figure 3:**
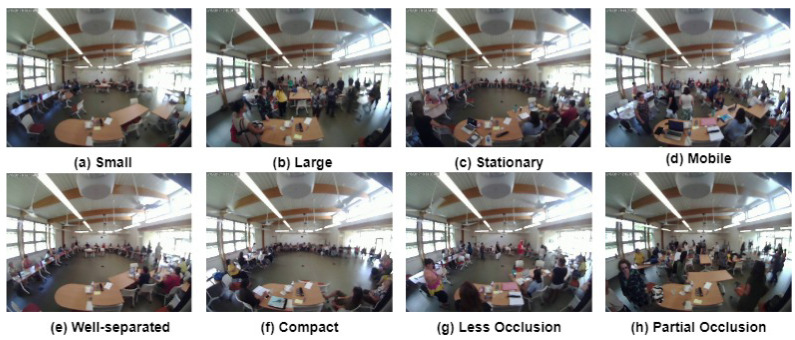
Different types of crowd found in FROG data.

The FROG video data recorded from 2017 to 2019 contains 432 hours of recordings. The videos were recorded from 9 am to 5 pm. There are several types of crowd (moderate, dense, sparse, moving, still, etc.) found in the data (Figure 3). The number of people present in the data varies from 0 to 31.

## 3. Methodology

**Figure 4:**
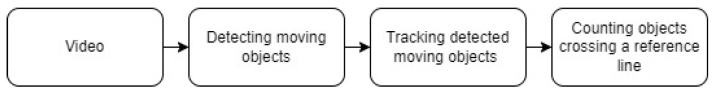
Motion-based occupancy counting system.

**Figure 5:**
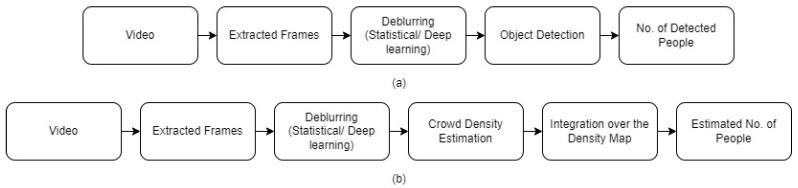
Motion-independent counting: (**a**) Object-detection-based occupancy counting framework, (**b**) crowd-density-estimation-based occupancy counting framework.

In this work, we propose two main approaches (Figure 3) for estimating occupancy from blurred video frames: motion-based and motion-independent techniques. Figure 4 illustrates the framework for the motion-based occupancy counting system, while Figure 5 depicts the framework for the motion-independent counting process.

### 3.1. Motion-Based Approach

The motion-based approach, as shown in Figure 4, consists of three main steps: detection of moving objects, tracking, and counting. To detect moving objects, we employ two methods: background subtraction and optical flow estimation. Background subtraction involves modeling the background using Gaussian mixture modeling (GMM) and subtracting it from the current frame to identify moving objects. Optical flow estimation, on the other hand, computes the motion vectors between consecutive frames using either sparse (Lucas–Kanade) or dense (Farneback) methods. After detecting the moving objects, we apply Kalman filtering to track their trajectories across frames. Finally, we perform counting by analyzing the trajectories and their intersection with a predefined reference line, as illustrated in figures in Section 4.3. By determining the direction of the trajectory relative to the reference line, we can increase or decrease the occupancy count accordingly.

### 3.2. Motion-Independent Approach

The motion-independent approach, as depicted in Figure 5, consists of two main stages: deblurring and counting. We propose two different deblurring methods to enhance the clarity of the blurred video frames: iterative statistical deblurring and learning-based deblurring using a U-Net architecture. The iterative statistical deblurring method is based on the Lucy–Richardson algorithm, but unlike the original approach, the blur radius is informed by the presence of blur in the image. The learning-based deblurring method utilizes a U-Net architecture trained on synthetically blurred crowd images to remove blur in an end-to-end fashion. After deblurring the video frames, we apply two different counting techniques: object-detection-based counting and crowd-density-estimation-based counting. For object-detection-based counting, we employ the aggregated channel feature (ACF) detector and a region-based convolutional neural network (Faster R-CNN) to localize and count individuals in the deblurred frames. In the crowd-density-estimation-based approach, we utilize a dilated convolutional neural network (CSRNet) to estimate the crowd density map, which is then integrated to obtain the occupancy count.

## 4. Motion-Based Detection and Counting

### 4.1. Detection of Moving Objects by Background Subtraction

**Figure 6:**
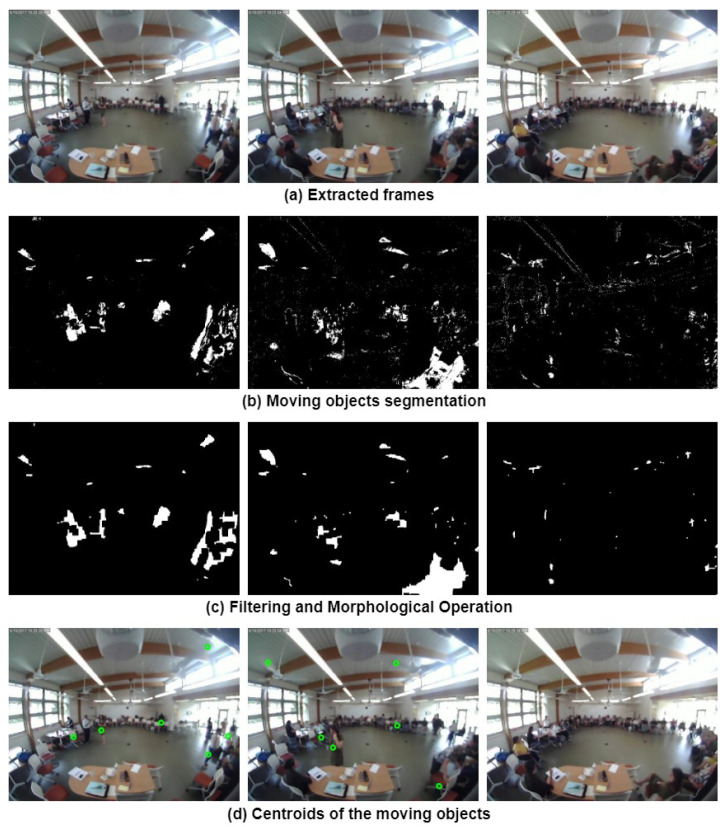
Different stages of background-subtraction-based moving object detection.

Real-time tracking and event analysis are two of many examples of computer vision applications that use foreground detection as the first step based on video streams. Foreground objects can be easily generated by using background modeling. Here, we used Gaussian mixture modeling (GMM) [37] to model each pixel in order to represent a dynamic background. GMM facilitates a robust detection system capable of handling issues [38] like movement in cluttered areas, overlapping objects, gradual and sudden illumination changes, slow-moving objects, and reflections from surfaces. These challenges are particularly relevant in our case, given the complex and dynamic nature of the surveillance video footage we are working with. The steps involving moving object detection are described below, and the corresponding results are shown in Figure 6.

#### Experiment

1. **Frame extraction:** Frames were extracted from the videos at a rate of six frames per second.
2. **Background modeling:** Gaussian mixture modeling (GMM) was used to model each pixel, representing the background as a mixture of *K* Gaussians. The probability of a pixel (x) belonging to the background was determined using the following equation:
  (1) $$p\left(x\right)=\sum_{k=1}^{K}\pi_{k}N\left(x|\mu_{k},\Sigma_{k}\right),\;\mathrm{subject}\;\mathrm{to}\;\sum_{k=1}^{K}\pi_{k}=1$$
  where $\pi_{k}$, $\mu_{k}$, and $\Sigma_{k}$ are the mixture weight, mean, and variance of the *k*th Gaussian component, respectively.
3. **Filtering:** Median filtering was applied to remove speckle noise. The median filter calculates the median of pixel values $\left(p_{j}\right)$ in a local neighborhood $\left(\Omega_{i}\right)$:
  (2) $${\bar{p}}_{i}=\mathrm{median}\left(p_{j}\right),\;j\in \Omega_{i}$$
4. **Morphological operations:** Morphological operations, (dilation (I⊕s), erosion (I⊖s), opening (I∘s) and closing (I·s)) were performed on the binary image (I) using a structuring element (s):
  (3) $$\mathrm{Dilation}:\;G\left(x,y\right)=\left\{\begin{array}{c} 1\;\mathrm{if}\;s\;\mathrm{hits}\;I,\\ 0\;\mathrm{otherwise} \end{array}\right.$$
  (4) $$\mathrm{Erosion}:\;G\left(x,y\right)=\left\{\begin{array}{c} 1\;\mathrm{if}\;s\;\mathrm{fits}\;I,\\ 0\;\mathrm{otherwise} \end{array}\right.$$
  (5) Opening:I∘s=(I⊖s)⊕s
  (6) Closing:I·s=(I⊕s)⊖s
5. **Blob detection:** Connected component labeling was used to identify connected components (blobs) in the binary image.
6. **Centroid calculation:** The centroid (c) of each blob was calculated using image moments:
  (7) $$c=\frac{1}{n}\sum_{i=1}^{n}x_{i}$$
  where $x_{i}$ are the points of the shape and *n* is the number of unique points. The centroid coordinates $\left(C_{x},C_{y}\right)$ were obtained using:
  (8) $$C_{x}=\frac{M_{10}}{M_{00}},\;C_{y}=\frac{M_{01}}{M_{00}}$$
  where $M_{pq}$ are the image moments.

### 4.2. Detection of Moving Objects by Optical Flow

The optical flow describes the direction and time pixels in a two-dimensional velocity vector, with the direction and velocity of motion assigned to a specific location in the image. We transfer the real-world three-dimensional time case to two-dimensional case to make computation simpler and faster. Using the 2-D dynamic brightness function of *I*, we may characterize the image in more detail, *I (x, y, t)*. Given that the change in brightness intensity does not occur in the motion field around the pixel, we may apply the following formula:

(9) I(x,y,t)=I(x+δx,y+δy,t+δt)

Then, if we apply Taylor series approximation on the right-hand side of Equation (1), and neglecting the higher-order terms we obtain

(10) $$\frac{\partial I}{\partial x}u+\frac{\partial I}{\partial y}v+\frac{\partial I}{\partial t}=0$$

Here,

$$\frac{\partial x}{\partial t}=u,\frac{\partial y}{\partial t}=v$$

**Figure 7:**
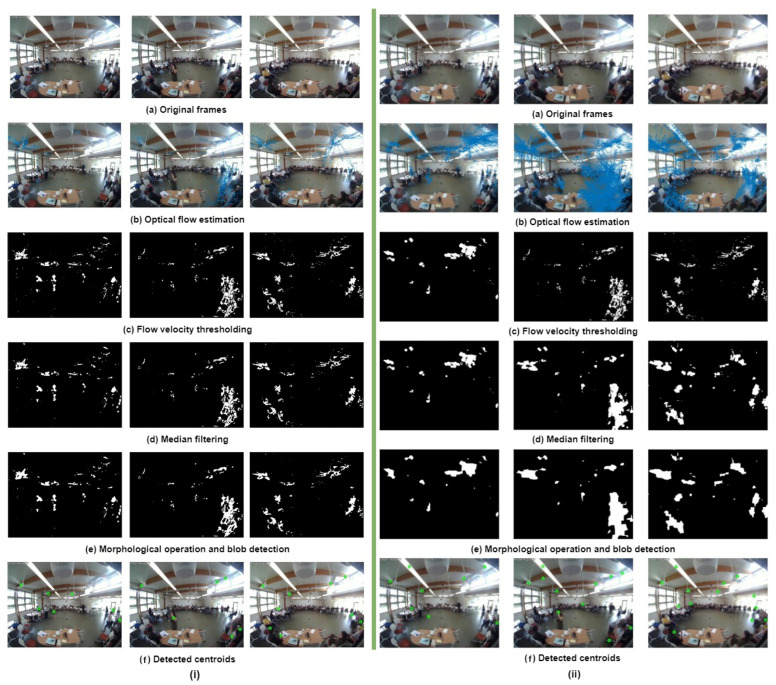
Different stages of optical-flow-based (**i**) sparse (Lucas–Kanade) and (**ii**) dense (Farneback) moving object detection.

$\frac{\partial I}{\partial x}$, $\frac{\partial I}{\partial y}$, and $\frac{\partial I}{\partial t}$ are the image gradients along the horizontal and vertical directions, and time. We need to solve u and v to determine the movement with time. As there is only one equation and two unknown variables, we cannot solve the optical flow equation in the usual manner. There are several sparse and dense optical flow methods to address this issue. Sparse flow methods compute velocity vectors for some sparse set of interesting features (edges, corners, etc.) and dense flow methods determine optical flow for all the pixels. The Lucas–Kanade method [39] is one of the most commonly used sparse flow approaches. This approach operates under the assumption that the motion vectors remain the same over a certain block of pixels and introduce an error term for each individual pixel. The smallest error can be computed by taking partial derivatives of the error term with respect to each component of velocity, and then, setting those partial derivatives equal to zero. On the other hand, the Farneback technique [40] is an example of a dense flow approach. It works by approximating the image window frames with a polynomial of degree 2, and the initial phase of the process involves expanding the polynomial. The The next step is to estimate the movement of fields by observing the transformation of the polynomial while the system is in the motion state. The calculation of dense optical flow is performed after a certain number of repetitions. In comparison to the sparse optical flow approach, the dense optical flow process is more time-consuming, but it produces more reliable results. In this work, both sparse and dense optical flow algorithms have been implemented to identify the foreground. The steps involving moving object detection through optical flow are described below and the corresponding results are shown in Figure 7.

#### Experiment

After extracting frames, both sparse and dense optical flow techniques were employed to detect moving objects. Subsequently, flow velocity thresholding was applied, which involves calculating the magnitude of optical flow from the x and y components of velocity in pixels per frame, as well as determining the mean velocity per frame. The square of the optical flow magnitude was then compared to the mean velocity to segment the moving objects.

Following the segmentation, filtering and morphological operations were performed to refine the detected objects. Blob detection was then applied to identify connected regions, and the centroids of these regions were calculated. These post-processing steps were carried out in the same manner as described in the background subtraction section.

### 4.3. Tracking and Counting

**Figure 8:**
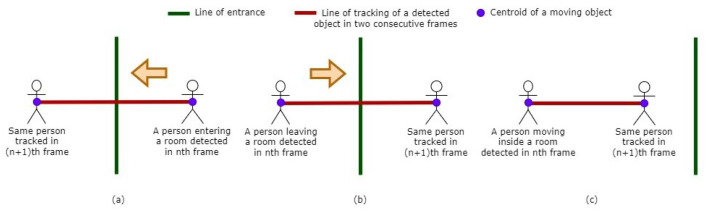
Reference-line-based counting: (**a**) occupant entering the room resulting increase in count, (**b**) occupant exiting the room resulting decrease in count, (**c**) occupant roaming inside the room resulting no change in count.

**Figure 9:**
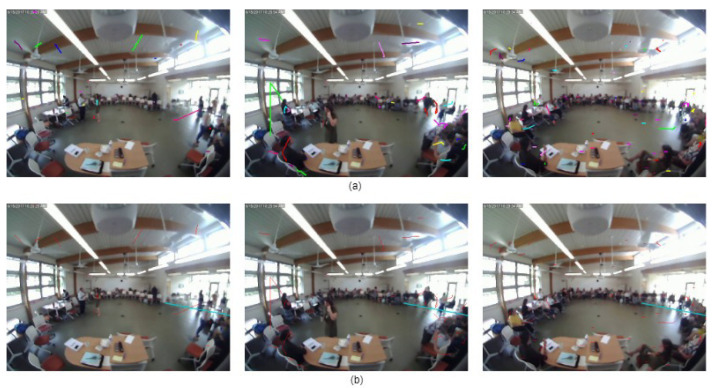
Tracking and counting objects: (**a**) tracks of detected moving objects; (**b**) checking whether an object crosses a reference line (cyan colored line).

Kalman filter [41] is used to track the centroids of the moving objects detected using the previously discussed methods. The linear motion of the objects and the computational efficiency of the Kalman filter make it the preferred choice over particle filters [42,43] for our tracking application. Then, for the purpose of counting, we propose a method (Algorithm 1) that leverages object centroid tracking across video frames to determine individuals’ movements in relation to a predefined reference line (Figure 8) within the field of view of a surveillance camera. By establishing a trajectory for each detected object between consecutive frames and examining its intersection with this line, we find out whether an individual has entered or exited the monitored space. The direction of movement determined by the relative positions of the object centroids across frames dictates the classification of movement as an entrance or exit. This counting method also excludes unwanted detected objects (e.g., ceiling fans) as it narrows the problem space to the entrance instead of the whole room. The process of counting is shown in Figure 9.
**Algorithm 1** Object Counting by Line Intersection**Input:** Reference line Ax+By+C=0, centroids in previous and current frames (Centroid 01 and Centroid 02)**for** each detected object **do**    Define line $A_{c}x+B_{c}y+C_{c}=0$ connecting Centroid 01 and Centroid 02    Calculate parameters $A,B,C,A_{c},B_{c},C_{c}$ for both lines    **if** $AB_{c}-A_{c}B\neq 0$ **then**        Find intersection (x,y) where $x=\frac{B_{c}C-BC_{c}}{AB_{c}-A_{c}B}$ and $y=\frac{AC_{c}-A_{c}C}{AB_{c}-A_{c}B}$        **if** $\min\left(x_{01},x_{02}\right)\leq x\leq \max\left(x_{01},x_{02}\right)$ & $\min\left(y_{01},y_{02}\right)\leq y\leq \max\left(y_{01},y_{02}\right)$ **then**           An object crosses the line. Determine direction:           **if** $x_{01}>x_{02}$ **then**               Object enters           **else**               Object exits           **end if**        **end if**    **end if****end for**

## 5. Motion-Independent Detection and Counting

### 5.1. Why Do We Need Deblurring?

The FROG video has been blurred by changing the focal length of the surveillance camera to ensure the privacy of the occupants present in a room. In the non-motion-based counting technique, we implement machine and deep learning algorithms. At first, we need to reduce the amount of blur present in the out-of-focus-induced blurred frames. The effects of blur on the performance of deep neural networks have been discussed in [44]. They showed that the deep neural architectures Caffe, VGG-16, VGG-CNN-S, and GoogleNet are very sensitive to the presence of blur and the networks’ performance deteriorates significantly even for moderate blur extents. The most probable reason is the removal of textures in images due to the smoothing effect caused by blur. Training the neural network with low-quality images is an apparent solution but the accuracy in the case of high-quality images might be affected. They also showed that VGG-16 is comparatively more resilient to the types and amounts of distortion than the other networks. Therefore, we added a deblurring stage instead of feeding the blurred frames directly to the counting stage.

### 5.2. Iterative Statistical Deblurring

A blurred image can be modeled using the following equation:

(11) B(x,y)=H(x,y)⊗G(x,y)+N(x,y)

where *(x, y)* represents spatial coordinates, B(x,y) is the blurred image, H(x,y) is the kernel or point spread function (PSF), G(x,y) is the sharp image, and N(x,y) is the additive noise. The PSF of the space-invariant or shift-invariant out-of-focus image can be described as in [42].

(12) $$H\left(x,y\right)=\left\{\begin{array}{cc} \frac{1}{\pi r^{2}} & \;\mathrm{if}\;{\left(x-m\right)}^{2}+{\left(y-n\right)}^{2}\leq r^{2},\\ 0 & \;\mathrm{elsewhere}\; \end{array}\right.$$

where *(m, n)* is the PSF center and r is the blur radius. The deblurring process in our case is blind, as we do not have any information about the radius of the blur kernel. In blind deblurring, we are given B(x,y) only, and our goal is to predict a latent image L(x,y), which is the closest approximation to the sharp image G(x,y). This is an ill-posed problem, as we have to predict both G(x,y) and H(x,y). In such a case, the deblurring technique is called blind deconvolution. Compared to blind deconvolution techniques, the Richardson–Lucy (RL) algorithm assumes that the blur kernel (PSF) is known. In our case, we chose the RL method as it is robust in the presence of noise, and it has been shown that the RL method performs better than the other classical deblurring methods [45].

####  Experiment

**Figure 10:**
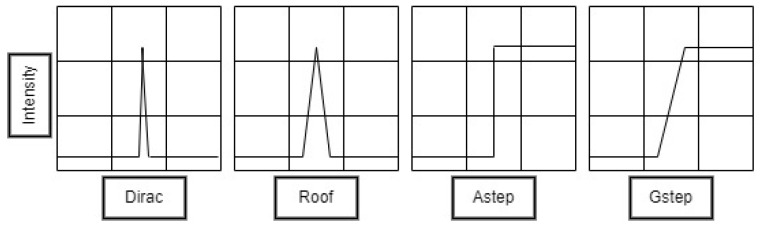
Types of edges found in natural images.

Since we are dealing with out-of-focus blur, the point spread function (PSF) is an airy disk. We empirically set the number of iterations to 100 and experimented with airy disks of 13 different radii, ranging from 1 to 7. For each airy disk, we applied the iterative RL algorithm to deblur the blurred frames. Instead of randomly selecting the radius of the blur kernel, we opted to choose the radius that yielded the least amount of blur in the deblurred frames. To achieve this, we employed a direct blur detection method [46] to calculate the blur extent in the deblurred frames. This approach allowed us to identify the optimal radius that minimized the residual blur after the deblurring process. In this method, Haar wavelet transform is used to decide whether an image is blurred or not by analyzing the edge types and the amount of blur present in the blurred image by analyzing the edge sharpness. There are four types of edges [46] found in natural images (see Figure 10): 1. Dirac structure, 2. Roof structure, 3. Astep structure, and 4. Gstep structure. The edges.

Both Dirac structures and Astep structures disappear when blur happens, regardless of whether it is generated by being out of focus or linear motion. Furthermore, both the Gstep structure and the roof structure tend to become less sharp in their structure [46]. This method decides whether a given image is blurred or not according to the presence of Dirac or Astep structures and evaluates the amount of blur by the percentage of Gstep structures and roof structures.

**Figure 11:**
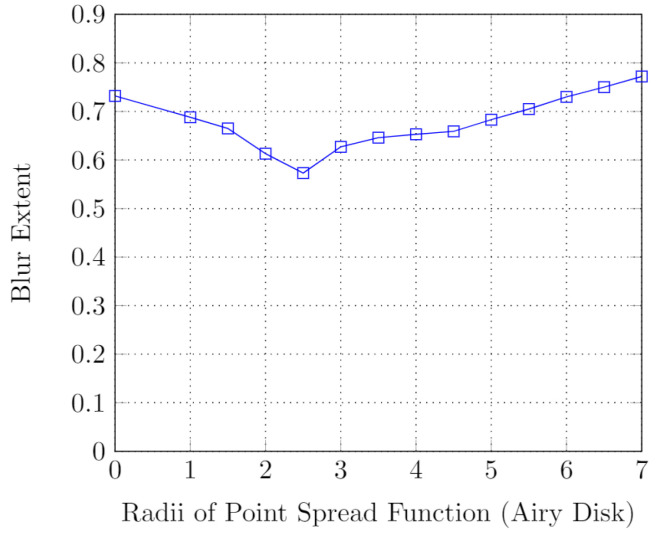
Amount of blur in deblurred image for different PSFs.

**Figure 12:**
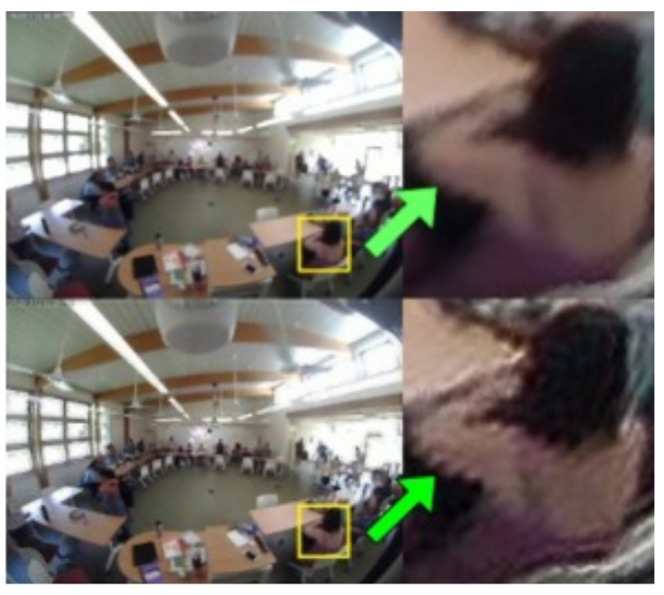
The original blurred FROG image (**top**) and the corresponding image deblurred by RL deblurring (**bottom**).

The deblurring algorithm combining the Lucy–Richardson method and the blur-extent calculation technique is shown by Algorithm 2 and an example is shown in Figure 11. We can see that the blur extent of a blurred frame is found to be minimum when the radius of the PSF is 2.5. Thus, we choose the deblurred frame corresponding to the PSF of radius 2.5 (Figure 12).
**Algorithm 2** Iterative Statistical Deblurring assisted by Blur-extent Calculation
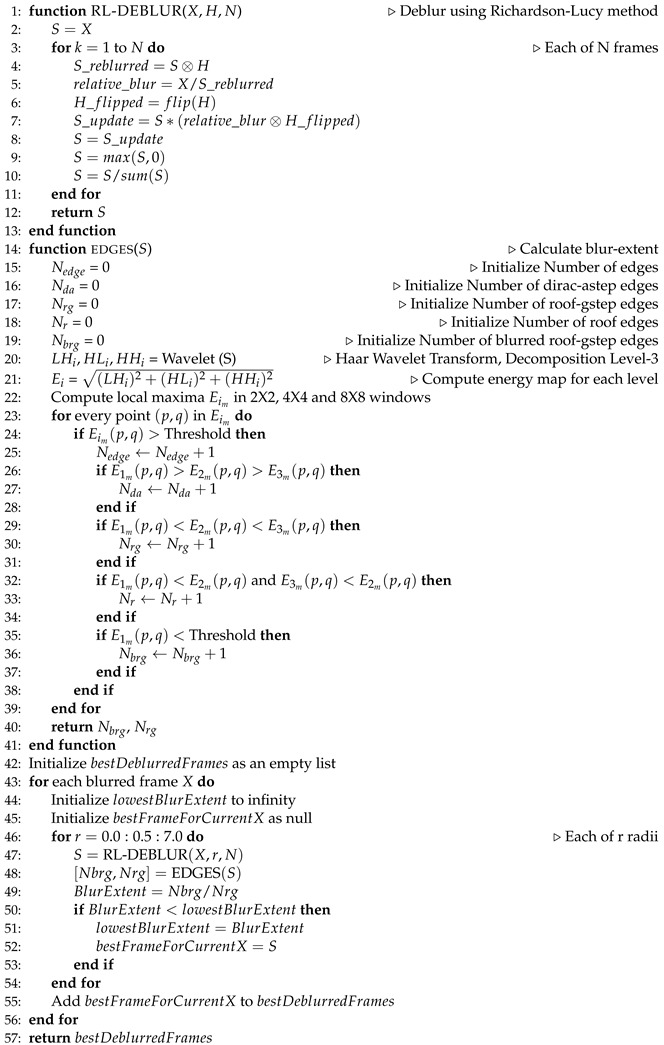


### 5.3. Learning-Based Deblurring

Deep learning has revolutionized image deblurring through diverse approaches, including end-to-end convolutional neural networks, generative adversarial networks [47], algorithm unrolling, learning in feature space, multi-scale processing, RAW image deblurring, and techniques for non-blind deblurring [48]. Each method leverages neural networks’ capabilities to restore sharpness from blurred images, enhancing image quality with state-of-the-art performance and offering unique advantages in tackling the complexity of deblurring tasks. In this work, we chose the U-Net [49] architecture for deblurring because of its ability to effectively manage multi-scale information [50], efficient feature fusion mechanism, and adaptability to various deblurring tasks [51].

The U-shaped architecture is made up of a certain encoder–decoder scheme, which is as follows: Every layer of the encoder decreases the spatial dimensions while simultaneously increasing the number of channels. The decoder, on the other hand, increases the spatial dimensions while simultaneously decreasing the number of channels. Bottleneck is the term used to describe the tensor that is fed into the decoder. The spatial dimensions are then restored, allowing an estimate to be made for each pixel in the input image at this point. ResNet-34 [52] is used as the encoder part of the U-Net, which is the backbone. This is to allow the use of a well-established image classification architecture with pre-trained weights for the purpose of transfer learning.

#### Experiment

**Figure 13:**
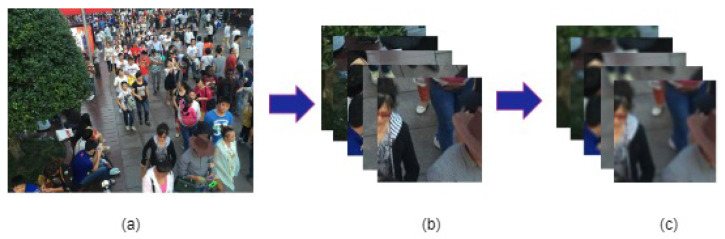
Training data preparation: (**a**) Sample image from Shanghaitech part B [53] crowd dataset; (**b**) extracted patches; (**c**) blurred patches.

In order to train the U-Net (hyperparameters shown in Table 2) we used the Shanghaitech part B [53] crowd dataset. We selected 375 images, each of which has a size 1024 × 768, and collected 24 patches of size 160 × 160 from each of the images. Thus, the total number patches was 9000. The patches were then divided into 90 groups randomly. Each group, consisting of 100 patches, was blurred by blurring disks of 90 different radii (1.00, 1.10, 1.15, …, 5.50). See Figure 13.

**Figure 14:**
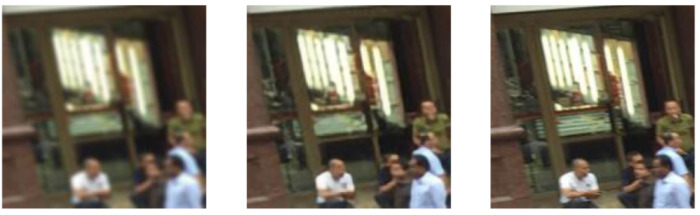
Blurred patch (**left**), patch deblurred by U-Net (**middle**), and original clean patch (**right**) of an image in Shaghaitech part B dataset.

We used perceptual loss combined with pixel mean squared error loss and gram matrix style loss [54,55]. We trained the U-Net for 100 epochs. The example of a predicted clean patch from a blurred patch along with the original clean patch is shown in Figure 14.

**Figure 15:**
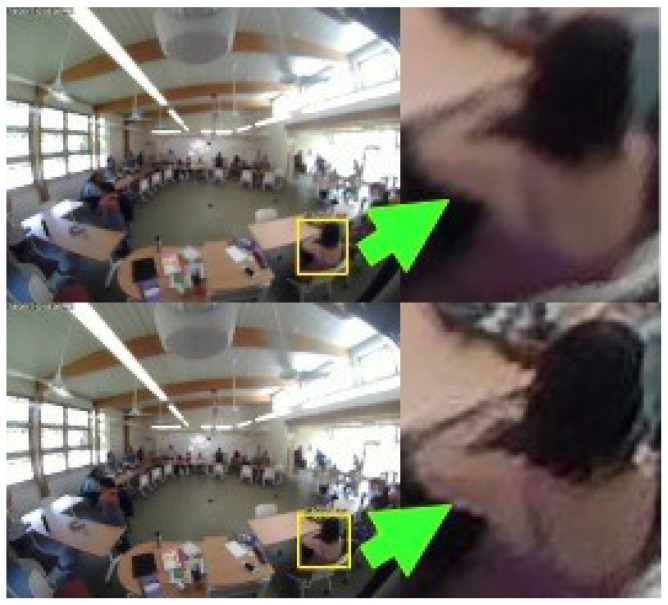
The original blurred FROG image (**top**) and the corresponding image deblurred by U-Net (**bottom**).

In order to deblur the FROG video frames, the frames are extracted from video captured through the surveillance camera for every one second. The resolution of the extracted frames is 640 × 480. Each frame is then divided into total 12 number of patches and each patch is then passed through the trained U-Net, resulting in an estimated deblurred patch. All of the deblurred patches are aggregated to give the final output, as shown in Figure 15.

### 5.4. Counting by Detection

#### 5.4.1. Aggregated Channel Features

The aggregated channel features (ACF) detector proposed in [56] has demonstrated good performance in a number of detection problems. In contrast to traditional channel features, aggregated channel features are extracted directly as pixel values in extended channels, rather than determining rectangular sums at different scales and locations, as is the case with traditional channel features. ACF consists of three LUV color channels, one normalized gradient magnitude channel, and six Histogram of Oriented Gradients (HoG) channels. An RGB image I(x,y) is first translated into the LUV color space, which is then followed by the calculations of the gradient magnitude and gradient orientation of image *I* using the following formulas, respectively.

(13) $$G_{M}\left(m,n\right)=\sqrt{{\left(\frac{\partial I(m,n)}{\partial x}\right)}^{2}+{\left(\frac{\partial I(m,n)}{\partial y}\right)}^{2}}$$

(14) $$G_{O}\left(m,n\right)=\tan^{-1}\left(\frac{\frac{\partial l(m,n)}{\partial y}}{\frac{\partial y(m,n)}{\partial x}}\right)$$

The gradient magnitude is smoothed by convolving it with a 2D triangle filter of the form $\frac{\left[1p1\right]}{2+p}$, where the value of *p* is calculated from the radius *r* of the triangle filter. Here, we used r=1. The smoothed gradient magnitude is then normalized using the following formula:

(15) $$\tilde{M}\left(m,n\right)=\frac{M(m,n)}{S\left(m,n\right)+n_{c}}$$

Here, S(m,n) is the smoothed gradient magnitude and $n_{c}$ is the normalization constant (0.005). After that, the histogram of oriented gradients (HoG) features are computed from the normalized gradient magnitude and gradient orientation with a cell size of 4 and six bins. All of the features are then aggregated and a decision tree is constructed.

##### Experiment

We collected 800 frames (positive training examples) which contained persons and 1643 frames (negative training examples) which did not contain any people from recorded videos of 10 different days. Both types of frames were deblurred using both the statistical method and the deep-learning-based method (RL and U-Net) as described in the deblurring stage. The hyperparameters used to train the ACF detector is shown in Table 3. In order to test the performance of the trained ACF detector, we extracted 100 frames from recorded videos of three different days and deblurred those in the same way as we deblurred the training images. The testing result is shown in Table 4. The performance is measured using the log-average missing rate (MR), where the missing rate is defined as follows:

(16) $$MR=\frac{FN}{TP+FN}$$

**Figure 16:**
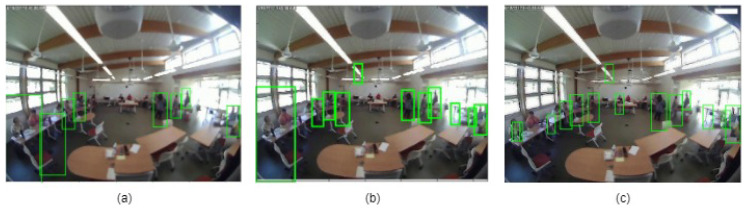
Detection results: (**a**) ACF detector, (**b**) U-Net+ACF detector, and (**c**) RL+ACF detector.

The detection results for the three different approaches are shown in Figure 16.

#### 5.4.2. Region-Based Convolutional Neural Network

There are three basic processes in traditional object detection methods. The first stage is to come up with a list of potential regions. There is a possibility that these regions might contain objects. Selective search and edge boxes are two examples of algorithms that produce regions. Then, a feature vector of fixed length is retrieved from each region proposal using several image descriptors, such as the histogram of oriented gradients (HoG). Object detectors rely on this feature vector to work properly. The vector should be able to accurately describe an object, regardless if it is scaled or translated. Using the feature vector, each region proposal is then assigned to one of the object or backgrounds classes using some classifier and classifying region proposals; using the support vector machine (SVM) is a common practice. Unlike traditional object detection techniques, the deep-neural-network-based approaches R-CNN [57] and Fast R-CNN [58] extract features based on just extracting the features based on a convolutional neural network (CNN). Faster R-CNN [59] is an enhancement of Fast R-CNN. The region proposal network makes Faster R-CNN faster than Fast R-CNN (RPN). Faster R-CNN consists of two modules: 1. Region Proposal Network (RPN) and, 2. Fast R-CNN. The RPN, a fully convolutional network, produces proposals of varied sizes and aspect ratios and introduces the idea of attention in neural networks, which guides the Fast R-CNN detection module to where to seek for objects in an image. The notion of anchor boxes was presented in the Faster R-CNN algorithm as an alternative to the traditional pyramids of pictures or filters. A reference box with a defined scale and aspect ratio is referred to as an anchor box. For a particular region, there are many reference anchor boxes with a variety of sizes and aspect ratios available. This can be considered to be a pyramid of reference anchor boxes. Each region is then mapped to each reference anchor box, resulting in the detection of objects with varying sizes and aspect ratios throughout the image.

##### Experiment

**Figure 17:**
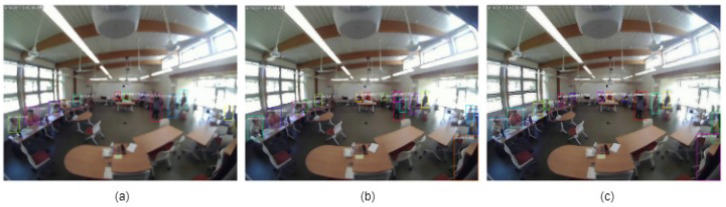
Detection results: (**a**) Faster R-CNN, (**b**) U-Net+Faster R-CNN, and (**c**) RL+Faster R-CNN.

There are three distinct methods for training both the RPN and Fast R-CNN while sharing the convolutional layers across the two networks: 1. Alternating training; 2. approximate joint training; and 3. non-approximate joint training. We used the alternating training method in which the RPN is initially trained how to develop region proposals. Deblurring (RL and U-Net) was performed on training, validation, and testing sets of 800, 200, and 100 frames, respectively. This was followed by bounding-box annotations of each person. The training dataset contained 8364 annotated persons, 956 annotated heads for validation, and 1063 annotated heads for testing. The hyperparameters used to train Faster-RCNN are shown in Table 5. The training and testing results are shown in Table 6. The detection results for the three different approaches using Faster R-CNN are shown in Figure 17. The calculation of the average precision (AP) is a weighted mean of the precision at each threshold, where the weight represents the increase in recall from the previous threshold. Precision and recall are defined as follows:

(17) $$\;\mathrm{Precision}\;=\frac{\;True\;Positive\;}{\;True\;Positive+\;False\;Positive\;}$$

(18) $$\;\mathrm{Recall}\;=\frac{\;True\;Positive}{\;True\;Positive\;+\;False\;Negative\;}$$

### 5.5. Counting by Density Map Estimation

#### Dilated Convolutional Neural Network

**Figure 18:**
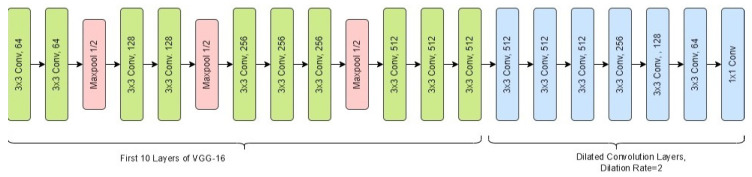
CSRNet architecture.

Detection-based counting approaches have limitations to performing well in the presence of occlusions (as we saw in the previous sections) and cluttered background. They also do not take spatial information into account. Lempitsky et al. [60] utilize spatial information in counting by modeling a density function as a linear combination of SIFT feature vectors, where integration of the density function over entire image gives the total count of objects. Pham et al. [61] introduce non-linearity as linear mapping poses difficulties. Both of these methods rely on hand-crafted features which result in less accurate counts. The following studies adopted CNN to estimate density more accurately as it does not depend on hand-crafted features. Zhan et al. [53] proposed MCNN, whose output is a density map. The integration gives the total number of heads. They used geometry-adaptive kernels to convert an image containing the labeled heads of people to a density map. Li et al. [62] show MCNN has structural redundancy and a larger amount of parameters are used for density map classification rather than density map generation, resulting in lower accuracy. They proposed CSRNet (Figure 18), which uses VGG-16 [63] as the front-end and dilated convolution layers as the back-end. It performs well for both densely crowded and sparsely crowded scenes. We used CSRNet to count the number of occupants.

#### Experiment

(i) Density map generation: Each image containing labeled heads is converted to a density map. A head at pixel $x_{i}$ is represented as a delta function $\delta (x-x_{i})$. Thus, the function representing *N* heads is

(19) $$H\left(x\right)=\sum_{i=1}^{N}\delta \left(x-x_{i}\right)$$

**Figure 19:**
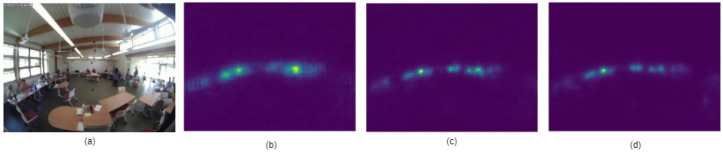
Occupants’ density maps estimated by CSRNet: (**a**) Original image, (**b**) CSRNet, (**c**) U-Net+CSRNet, and (**d**) RL+CSRNet.

We need to convolve H(x) with a Gaussian kernel of variance σ, $G_{\sigma }$ to make H(x) a continuous density function. The variance should be made dependent of each head size in the image to reduce the effect of perspective distortion caused by the homography between image and ground plane. Therefore, the variance is defined as

(20) $$\sigma_{i}=\beta {\bar{d}}^{i}$$

where ${\bar{d}}^{i}=\frac{1}{m}\sum_{j=1}^{m}d_{j}^{i}$ is the average distance between a head and its *k*-nearest neighbors and β is empirically found to be 0.3. The resultant continuous density function is [38]

(21) $$F\left(x\right)=\sum_{i=1}^{N}\delta \left(x-x_{i}\right)*G_{\sigma_{i}}\left(x\right)$$

(ii) Training and testing: In order to train the network (Figure 18), we chose the hyperparameters shown in Table 7. The training, validation, and test datasets consisted of 800, 200, and 100 frames, respectively, which were deblurred first using both statistical and deep-learning-based methods (RL and U-Net), as described in the deblurring stage. Then, we annotated the heads of the occupants and generated density maps in the process described in the density map generation stage. There are a total of 8364 annotated heads in the training dataset, 956 annotated heads in the validation dataset, and 1063 annotated heads in the testing dataset. The training and testing results are shown in Table 8. The estimation results for the three different approaches using CSRNet are shown in Figure 19. To evaluate the performance of the model, we used mean absolute error (MAE) as the metric, which is defined as

(22) $$MAE=\frac{1}{N}\sum_{1}^{N}\left|C_{i}-{\hat{C}}_{i}\right|$$

where *N* is the number of test images, $C_{i}$ is the original count, and ${\hat{C}}_{i}$ is the estimated count, which is defined as

(23) $${\hat{C}}_{i}={\int \int }_{S}F\left(X_{i};\Theta \right)dxdy$$

Here, S is the spatial region estimated by the trained network.

## 6. Results and Discussion

### 6.1. Performance Comparison

We compare the performance of different motion-based and non-motion-based methods to count the number of occupants, as shown in Table 9. In order to evaluate the performance of detection-based counting we used the counting error, which is defined as follows:

(24) $$Counting\;Error=\sum_{t=1}^{N}\left|\frac{{\mathrm{Observed}}_{t}-\;{\mathrm{Predicted}}_{t}}{{\mathrm{Observed}}_{t}}\right|\times \frac{100}{N}$$

**Figure 20:**
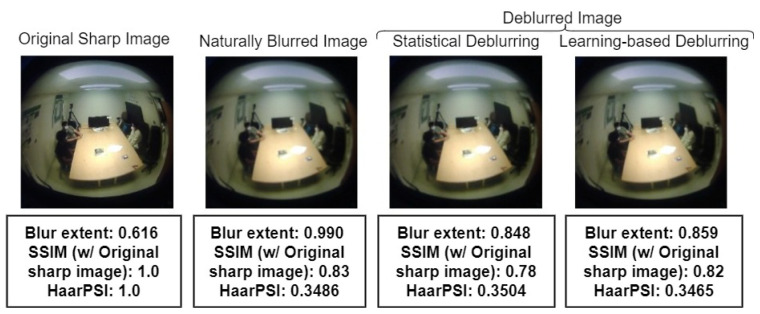
Comparing blur extent, SSIM, and Haar PSI of original (ground truth), blurred, and deblurred images.

**Figure 21:**
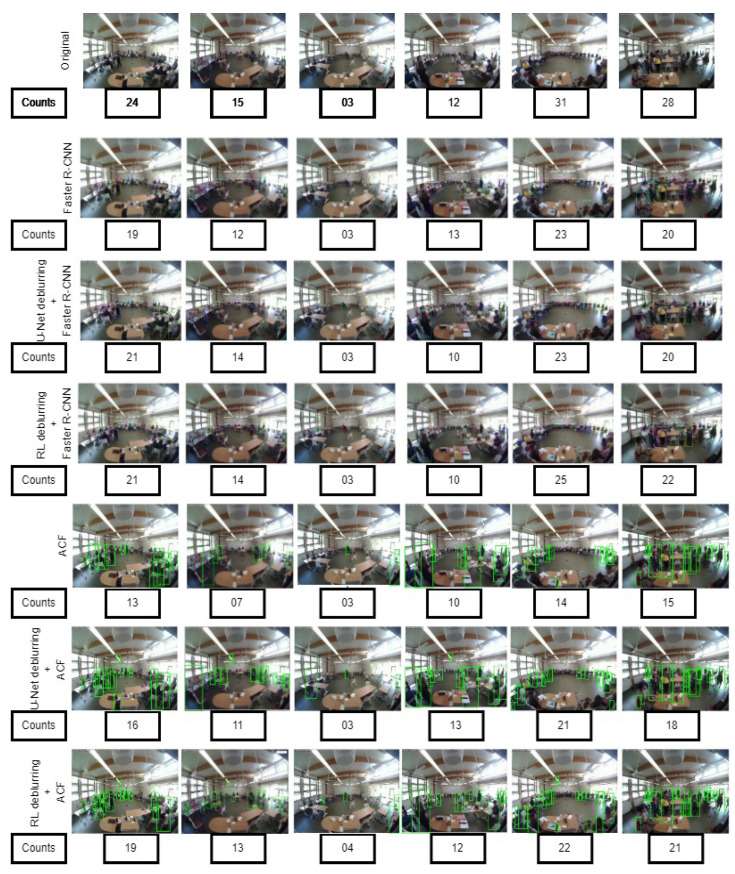
Number of occupants estimated by different object detection methods.

Here, N = total number of frames. From Table 9 we can see that the motion-based techniques perform the worst. The change in illumination between the doorway and indoors, the complex and cluttered background, and shadows of the occupants affect the performance of background subtraction. On the other hand, the sparse optical flow method faces difficulties in detecting the objects moving fast. Dense optical flow tries to solve the problem to some extent and provides better results compared to the sparse method. The background subtraction and optical flow estimation methods both suffer from motion discontinuities caused by faster moving objects but slower frame rates, and most importantly occlusions when a moving occupant occludes another moving occupant. The performance of the motion-based counting method may be improved by placing the camera right above the doorway. Overall non-motion-based approaches provide better counting accuracy than the motion-based approaches. In the case of non-motion-based approaches, Table 4 and Table 8 show that both Richardson–Lucy (RL) and U-Net-based deblurring methods improve the performance of the detection-based ACF (missing rate) and density map-based CSRNet (mean absolute error). On the contrary, the average precision (AP) of Faster R-CNN decreases (Table 6) slightly after the introduction of the deblurring methods. In fact, the texture and details in the frames are increased by deblurring methods, which contributes to the increase in both true and false positive cases. Thus, although the precision of Faster R-CNN might be negatively affected, the recall is improved at the same time. In all (Table 4, Table 6, and Table 8) approaches it can be seen that U-Net provides inferior results compared to the Richardson–Lucy deblurring. The reason is that instead of training the U-Net with images blurred by a particular point spread function, we have trained it with images blurred by point spread functions of different radii (90) to make U-Net suitable for the blind deblurring process. The frames with a higher blur extent affect the frames with a lower blur extent. The overall performance of U-Net, thus, is affected. The overall performance of ACF detection is poor as it suffers from an occlusion problem in crowded scenes and the hand-crafted features are not enough to detect objects. On the other hand Faster R-CNN performs better compared to the ACF detector as its deep neural network architecture is capable of extracting features to detect objects even in challenging scenes. Density-map-based approaches are capable of reducing counting errors caused by occlusions as the methods put spatial information into use. The CSRNet captures high-level features by utilizing larger receptive fields and produces high-quality density maps without significantly increasing network complexity. It can be seen from Table 8 that CSRNet alone performs poorly in detecting objects from blurred video frames. The application of Gaussian blur kernels on already blurred video frames makes it difficult for CSRNet to extract features. The performance improves when the deblurring stage is incorporated. The performance of different motion-independent occupancy estimation approaches is shown in Figure 20 and Figure 21.

### 6.2. Deblurring and Privacy

Quantifying visual privacy in images or videos is an open research problem, and there is a lack of standard metrics for this purpose. Developing such metrics is beyond the scope of our current work. However, to address the privacy implications of our occupancy counting system, we conducted an analysis using three proxy measures: blur extent [46], structural similarity (SSIM) [64], and perceptual difference (HaarPSI) [65]. These metrics, while not directly measuring privacy, provide insights into the quality and perceptual differences between the original, blurred, and deblurred frames.

#### 6.2.1. Blur Extent

Blur extent is a measure of the amount of blur present in an image. We used a direct blur detection method based on the Haar wavelet transform to estimate the blur extent. This method analyzes the edge types and edge sharpness in the image to determine the degree of blur. A higher blur extent indicates a greater level of privacy protection, as the visual details are more obscured.

#### 6.2.2. Structural Similarity (SSIM)

The structural similarity (SSIM) index is a widely used metric for assessing the perceived quality of an image. It measures the similarity between two images based on three factors: luminance, contrast, and structure. SSIM values range from 0 to 1, with higher values indicating greater similarity between the images. In the context of privacy analysis, a lower SSIM value between the original and deblurred frames suggests a higher level of privacy protection, as the deblurred image differs more from the original.

#### 6.2.3. Perceptual Difference (HaarPSI)

The Haar Perceptual Similarity Index (HaarPSI) is a metric that quantifies the perceptual difference between two images. It is based on the Haar wavelet transform and considers the human visual system’s sensitivity to changes in different frequency bands. A higher HaarPSI value indicates a greater perceptual difference between the images, implying a higher level of privacy protection.

#### 6.2.4. Analysis

**Synthetically blurred dataset**

It was difficult for us to measure the amount of degradation left after deblurring, as we do not have the original sharp FROG images to compare with. Hence, we opted to evaluate the change in blur extent by comparing images from a comparable dataset before and after the application of deblurring techniques. We chose the Shanghaitech-Part B crowd dataset for this purpose. In our analysis, the average blur amount in sharp images was measured at 0.405. After introducing synthetic blur, we applied two deblurring techniques: statistical deblurring, which achieved a blur extent of 0.453; and deep-learning-based deblurring, with a blur extent of 0.494. Both methods resulted in images that were still blurrier than the original, sharp images.

**Naturally blurred dataset**

We also wanted to investigate the effect of deblurring on naturally blurred images. We could not use the FROG data as it does not have the sharp video frames as ground truth. Therefore, we recorded occupancy-related data from our lab with the permission of the participants. Then, we blurred the video by changing the focal length of the video camera (natural blur) and deblurred the video frames using both statistical and deep-learning-based deblurring.

**Figure 22:**
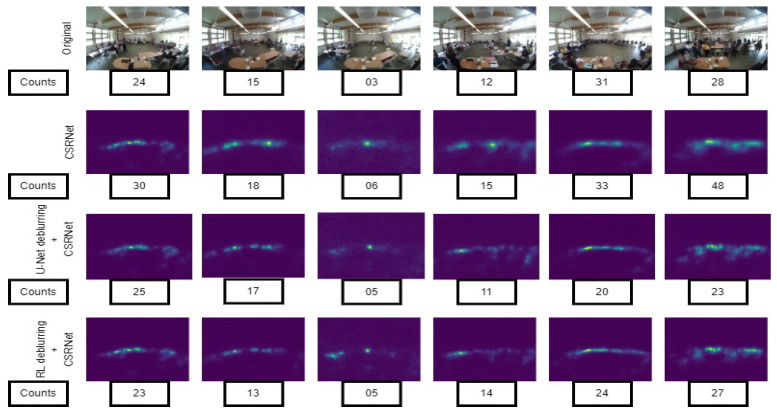
Number of occupants estimated by density estimation method.

In Figure 22, it is evident that the blur extent in the deblurred images produced by both statistical and deep-learning-based methods remains higher than that of the original sharp image. The statistical deblurring method relies on simplified parametric forms to model the point spread function (PSF), which often fails to accurately represent natural blur as it is difficult to estimate [66]. The mismatch between the modeled and actual PSFs can result in artifacts and suboptimal deblurring [67], explaining the persistence of blur even after the deblurring process. Similarly, training the U-Net in an end-to-end manner using images blurred with various blur kernels may contribute to the presence of residual blur following the learning-based deblurring [68].

The deblurred images are also structurally different to the original sharp image, which is indicated by the SSIM. Because of the ringing artifacts, the statistically deblurred image has a lower SSIM. Moreover, the HaarPSI index [58] shows that the deblurred images are also perceptually different from the original sharp image. Based on the blur extent, SSIM, and HaarPSI, we can say that there is still degradation present in the deblurred images to some extent, which ensures that the visual privacy of the occupants has not been completely compromised.

The presence of residual blur and artifacts in the deblurred images suggests that some level of privacy is still maintained. The higher the blur extent and the lower the SSIM and HaarPSI values, the more likely it is that the deblurred images preserve a certain degree of privacy compared to the original sharp images.

While these metrics serve as proxy measures for privacy, they do not directly quantify the level of privacy protection. To obtain a more direct measure of the relationship between deblurring and privacy, conducting a user study or perceptual evaluation could be beneficial. Participants could be asked to rate the level of privacy or the ability to identify individuals in the deblurred images compared to the original sharp images.

We acknowledge the limitations of our current approach and highlight the need for future research to develop more robust and standardized metrics for quantifying visual privacy in images and videos.

## 7. Conclusions

Occupancy estimation from blurred video while preserving the subjects’ privacy is a challenging task. To address this issue, we employed both motion-based and motion-independent algorithms. Although background-subtraction- and optical-flow-estimation-based counting methods can reliably detect motion despite the low resolution of the video frames, their accuracy is limited by their inability to detect occluded and fast-moving objects, as well as varying lighting conditions. As motion-based occupancy counting methods inherently ensure privacy, addressing these limitations could be beneficial for privacy-concerned occupancy counting research.

Motion-independent approaches, on the other hand, generally deliver better performance. Detection-based methods, such as the ACF detector and Faster R-CNN, struggle in crowded environments and scenarios with significant activity, resulting in substantial counting errors. In contrast, the density-map-based algorithm CSRNet reduces counting errors by minimizing the effects of occlusion. For both detection-based and density-map-based methods, statistical and deep-learning-based deblurring techniques improve counting performance while preserving occupants’ privacy to some extent, with the statistical method outperforming the deep learning approach. Consequently, the combination of statistical deblurring and density estimation yielded the lowest counting error.

Future research could focus on quantifying visual privacy and developing metrics to measure the impact of deblurring on privacy. This would provide valuable insights for designing occupancy counting systems that strike a balance between accuracy and privacy preservation. 

## Figures and Tables

**Table 1:** Comparison between existing and proposed occupancy estimation approaches.

Types		Existing Approaches	Proposed Approaches
Motion-dependent		• Multiple sensors need to be installed for a multi-entrance room. • Readings from multiple sensors need to be coordinated for total count for a multi-entrance room. • Difficulty in distinguishing between multiple people who are close together or moving as a group.	• A single camera can cover multiple entrances. • Properly placed camera can identify individual people moving in a group.
Motion-independent	Sound, Temp., Humidity, Pressure, CO_2_	• Sensors of diverse parameters must be coupled. • Build-up or dispersion rate might be affected by external factors.	• A single camera is used. • Does not depend on changes in environmental characteristics.
	Wi-Fi, Bluetooth, RFID	• Occupants must have their devices turned on.	• Does not depend on occupants’ inputs.
	Thermal camera	• Challenging to distinguish between individuals who are close together due to low spatial resolution.	• Visible light camera has higher resolution than thermal imaging camera.
	Camera	• Deals with small and well-separated occupants. • Privacy issue has not been addressed. • Multiple cameras need to be installed for a multi-entrance room.	• Deals with versatile crowd types (Figure 3). - Privacy issue has been addressed. • A single camera is used.

**Table 2:** Hyperparameters for U-Net.

Parameter	Value
Batch size	8
Learning rate	0.001
Epochs	300
Weight decay	0.001
Optimizer	SGD
Momentum	0.90

**Table 3:** Hyperparameters for ACF.

Parameter	Value
Maximum depth of tree	2
Number of stages	3
Number of weak classifiers	[32,64,128]
Maximum number of negative windows to sample	5000

**Table 4:** Performance of ACF.

Algorithms	Dataset	MR
ACF	Test	31
U-Net+ACF	Test	28
RL+ACF	Test	26

**Table 5:** Hyperparameters for Faster R-CNN.

Parameter	Value
Batch size	8
Learning rate	0.001
Epochs	500
Weight decay	0.0001
Optimizer	SGD
Momentum	0.90

**Table 6:** Performance of Faster R-CNN.

Algorithms	Dataset	AP
Faster R-CNN	Validation	0.758
	Test	0.743
U-Net+Faster R-CNN	Validation	0.750
	Test	0.732
RL+Faster R-CNN	Validation	0.753
	Test	0.736

**Table 7:** Hyperparameters for CSRNet.

Parameter	Value
Batch size	8
Learning rate	0.001
Epochs	400
Weight decay	0.0005
Optimizer	SGD
Momentum	0.95

**Table 8:** Performance of CSRNet.

Algorithms	Dataset	MAE
CSRNet	Train	3.682
	Validation	3.927
	Test	4.536
U-Net+CSRNet	Train	1.654
	Validation	1.867
	Test	2.014
RL+CSRNet	Train	1.691
	Validation	1.785
	Test	1.813

**Table 9:** Performance comparison of different approaches.

Approache	Algorithms	Counting Error (%)
Motion-based	Background Subtraction+Kalman Filter Tracking+Detection of Line Crossing	48.14
	Optical Flow Estimation (Sparse)+Kalman Filter Tracking+Detection of Line Crossing	46.24
	Optical Flow Estimation (Dense)+Kalman Filter Tracking+Detection of Line Crossing	44.73
Motion-independent	ACF	28.87
	RL+ACF	24.54
	U-Net+ACF	26.14
	Faster R-CNN	22.72
	RL+Faster R-CNN	19.95
	U-Net+Faster R-CNN	20.21
	CSRNet	31.28
	RL+CSRNet	16.29
	U-Net+CSRNet	18.24

## Data Availability

The data presented in this study may be available on request from the corresponding author. The data are not publicly available due to their proprietary nature.
